# Literature Review and Comparison of Two Statistical Methods to Evaluate the Effect of Botulinum Toxin Treatment on Gait in Children with Cerebral Palsy

**DOI:** 10.1371/journal.pone.0152697

**Published:** 2016-03-31

**Authors:** Angela Nieuwenhuys, Eirini Papageorgiou, Todd Pataky, Tinne De Laet, Guy Molenaers, Kaat Desloovere

**Affiliations:** 1 Department of Rehabilitation Sciences, KU Leuven, Leuven, Belgium; 2 Clinical Motion Analysis Laboratory, University Hospitals Leuven, Leuven, Belgium; 3 Department of Bioengineering, Shinshu University, Ueda, Japan; 4 Faculty of Engineering Science, KU Leuven, Leuven, Belgium; 5 Department of Development and Regeneration, KU Leuven, Leuven, Belgium; 6 Department of Orthopedics, University Hospitals Leuven, Leuven, Belgium; Institute Pasteur, FRANCE

## Abstract

**Aim:**

This study aimed at comparing two statistical approaches to analyze the effect of Botulinum Toxin A (BTX-A) treatment on gait in children with a diagnosis of spastic cerebral palsy (CP), based on three-dimensional gait analysis (3DGA) data. Through a literature review, the available expert knowledge on gait changes after BTX-A treatment in children with CP is summarized.

**Methods:**

Part 1—Intervention studies on BTX-A treatment in children with CP between 4–18 years that used 3DGA data as an outcome measure and were written in English, were identified through a broad systematic literature search. Reported kinematic and kinetic gait features were extracted from the identified studies. Part 2—A retrospective sample of 53 children with CP (6.1 ± 2.3years, GMFCS I-III) received 3DGA before and after multilevel BTX-A injections. The effect of BTX-A on gait was interpreted by comparing the results of paired samples t-tests on the kinematic gait features that were identified from literature to the results of statistical parametric mapping analysis on the kinematic waveforms of the lower limb joints.

**Results:**

Part 1–53 kinematic and 33 kinetic features were described in literature. Overall, there is no consensus on which features should be evaluated after BTX-A treatment as 49 features were reported only once or twice. Part 2—Post-BTX-A, both statistical approaches found increased ankle dorsiflexion throughout the gait cycle. Statistical parametric mapping analyses additionally found increased knee extension during terminal stance. In turn, feature analyses found increased outtoeing during stance after BTX-A.

**Conclusion:**

This study confirms that BTX-A injections are a valuable treatment option to improve gait function in children with CP. However, different statistical approaches may lead to different interpretations of treatment outcome. We suggest that a clear, definite hypothesis should be stated a priori and a commensurate statistical approach should accompany this hypothesis.

## Introduction

Pathological gait is one of the most striking characteristics in children with cerebral palsy (CP) [1]. When spasticity, weakness or other CP-related motor impairments manifest during walking, they may significantly restrict patients at the level of ‘activities’ and ‘participation’ of the International Classification of Functioning, Disability and Health [2]. There is a wide variety of treatments which can be applied to improve gait, ranging from conservative treatments such as physiotherapy, orthotics, and Botulinum toxin A (BTX-A) injections to surgical interventions such as single event multilevel surgery and selective dorsal rhizotomy. Depending on a patient’s symptoms and age, different treatment modalities might be appropriate.

Gait changes after treatment are often objectively quantified by using three-dimensional gait analysis (3DGA). 3DGA provides a large amount of multivariate kinematic and kinetic waveforms, which have proven to be highly valuable during the clinical decision-making process[3], [4]. Researchers and clinicians are challenged to extract and analyze the clinically relevant information from this large amount of data.

In general, specific, directed hypotheses are not stated prior to data collection. For example, a typical null hypothesis may state that there are no differences between the knee and ankle kinematics of children with CP pre- and post-treatment. As a consequence, the full gait cycles of the knee and ankle joints should ideally be considered in statistical analysis because the hypothesis implicitly pertains to the full gait cycle and to all knee/ankle kinematic variables.

In the literature however, several intervention studies that have examined the effect of treatment on gait, have reduced the amount of 3DGA data a priori by analyzing a number of specific kinematic or kinetic gait features, which refer to a specific time instant of a gait cycle, e.g. peak values [5–9]. The rationale behind the selection of these features is often unclear because those specific variables rarely appear explicitly in hypotheses. Most likely, they were chosen based on available clinical expert knowledge, through literature search, or potentially after viewing the data. Depending on the available clinical expertise, reducing this large amount of data a priori or post-hoc may introduce bias and potential clinically relevant information could be overlooked. Furthermore, by conducting statistical testing on multiple dependent gait features in relatively small sample sizes, the risk of detecting a false positive outcome (type I error) increases. In turn, a Bonferroni correction, which is often applied to deal with this risk, will increase the probability of obtaining a false negative result (type II error)[10].

In the past years, a promising approach called statistical parametric mapping (SPM) was introduced to the field of biomechanics [11], [12]. SPM is a statistical method able to perform hypothesis testing on kinematic and kinetic data in a continuous manner, thereby making a priori data reduction for non-directed hypotheses redundant. Additionally, it also takes into account the dependency between different time instances of the gait cycle [11], [12]. SPM has already been used for example to evaluate whether electromyography (EMG) time-series of four lower limb muscles during the stance phase are different between children and adults [13]. So far, it has not yet been used to evaluate the outcome of treatment in CP.

After a thorough search of the available literature, following the inclusion criteria described in the methods section of this paper, 223 peer reviewed scientific papers that evaluated the effect of treatment on gait in children with CP based on the analysis of kinematic or kinetic gait features, were identified. Some of the gait features that were analyzed in these papers to quantify the effect of treatment on gait were recurrent in many studies, while some others were only reported a small number of times. Furthermore, it appeared that the definitions of several features were somewhat unclear, making it difficult for researchers to reproduce or confirm the results. An example is the feature ‘hip extension during terminal stance’. It is not clear whether this feature refers to a specific time instance of the gait cycle, or whether it refers to the mean value or peak value of the hip during a phase of gait. Furthermore, the phase ‘terminal stance’ could potentially be defined differently across various studies because patients presenting with a pathological gait might not have a typical 60/40 ratio for stance and swing phase or might not display a typical heel rise in case they do not reach a flat foot position[14], [15].

To assess the value of SPM analysis as an alternative approach to feature analysis with regard to the interpretation of treatment outcome based on 3DGA in children with CP, this study will focus on one treatment modality, namely BTX-A injections. BTX-A is used to treat spasticity and has been proven to improve function and delay deterioration towards fixed muscle contractures or bony deformities[16], [17]. After tone reduction, 3DGA can highlight a child’s ability to alter their gait pattern. It can also detect to what extent other clinical motor symptoms such as postural instability or muscle weakness may contribute to the pathological gait pattern [18].

The aim of this study was twofold. First, the available expert knowledge on gait pattern changes in children with CP after BTX-A treatment was summarized. By performing a systematic literature search, an overview of gait features that have been frequently reported in literature and that have been shown to be responsive to BTX-A treatment was created. Secondly, the effect of multi-level BTX-A treatment on gait in a retrospective sample of children with CP was evaluated by comparing the results of the frequently reported feature analysis to the results of SPM analyses on the kinematic waveforms of the lower limb joints. It was expected that SPM would be judged as a valuable alternative statistical approach to describe the effect of BTX-A on gait in a wider and more unbiased perspective than the traditional feature analysis.

## Material and Methods

Ethical approval for this project was granted by the Medical Ethical Committee of University Hospitals Leuven, reference s56036. All patient information was anonymized prior to statistical analysis. Two major methodological parts were related to the main study goals. The first part involved a literature search to define and select reported gait features that quantify the effect of BTX-A treatment. The second part was an experimental outcome study, comparing SPM analysis to feature analysis in order to interpret the outcome of 3DGA pre- and post-BTX-A treatment in a group of children with CP.

### Literature search

A broad systematic search in the databases of Pubmed, Embase, Cinahl, and Web Of Science was performed. Key words included cerebral palsy, diplegia, hemiplegia, quadriplegia, gait analysis, locomotion, walking, gait, feature, parameter, variable, and characteristic. Relevant wildcard symbols were used to ensure all key word variations were searched and if possible, searches were limited to human studies, age, and language. After removal of duplicates, references were screened based on title and abstract. Eligibility of full-text papers was then assessed based on the following inclusion criteria: (a) intervention studies evaluating the effect of treatment on gait, using instrumented 3DGA; (b) the experimental population consisting for at least 80% out of children between the ages of 4–18 years with a diagnosis of the spastic type of CP; (c) a definition of kinematic and/or kinetic features, including at least joint angles or moments; (d) English full text availability in a Belgian library or at request to the author. We excluded case series, literature reviews, and intervention studies that only reported indices (such as GPS, GGI, etc.), EMG features, spatio-temporal parameters or children with dystonia.

The literature search explored all papers that have reported any type of treatment modality to improve gait in children with CP, using 3DGA as an outcome measurement tool. For the purpose of this study, only intervention studies evaluating BTX-A treatment were selected. All kinematic and kinetic features of the papers identified during the review, were extracted. Subsequently, features with different terminology were grouped together in case they had a similar meaning (e.g. minimal hip angle in sagittal plane during stance and maximum hip extension during the gait cycle). Apart from the initial literature search, two reviewers completed each step of the review process and a third reviewer was consulted in case of disagreement. The first search was conducted on December 9, 2013 and it was updated on October 28, 2015.

### Experimental outcome study

#### Patients and treatment characteristics

Patients were retrospectively selected from the database of the Clinical Motion Analysis Laboratory of University Hospital Pellenberg, Leuven, Belgium. We considered children with CP who attended the hospital for BTX-A treatment between 2004 and 2014. Children eligible for this study met following inclusion criteria: (a) age between 4–18 years, (b) a predominantly spastic diagnosis of CP, (c) walking with or without assistance of aids (GMFCS I-III), (d) BTX-A injections had occurred in at least hamstrings and gastrocnemius muscles (e) a maximum of three months between the date of BTX-A injection and the pre-3DGA, (f) at least 1 month and maximally 4 months between the date of BTX-A injection and the post-3DGA. Exclusion criteria were: symptoms of dystonia or ataxia and previous orthopedic surgery.

In case multiple gait analysis sessions were available for one patient, a preference was given to the gait analyses that were collected before and after the first or second BTX-A treatment because patients are likely to improve more after the first treatments [19]. All BTX-A injections were performed by a pediatric orthopedic surgeon and were part of an integrated multilevel treatment approach which was previously described by Molenaers et al[20]. BTX-A injections were administered under general (mask) anesthesia, applying a dilution of 100 units (U) of Botox® (Allergan, Inc. Irvine, USA) in 5ml of saline. The muscles selected for treatment were injected at multiple sites with a maximum of 50U per site and a minimal distance of 5 cm between injection sites. In accordance with the integrated approach, serial stretching casts, an increased number of physiotherapy sessions, and increased use of orthoses were planned after BTX-A treatment.

#### Data collection procedure

All gait analyses were conducted in the clinical motion analysis laboratory of the University Hospital Pellenberg, using a Vicon system (Oxford Metrics, Oxford, UK) and two AMTI force plates (Advanced Mechanical Technology Inc., Watertown, MA, USA). Children were asked to walk barefoot on a 10m walkway at a self-selected, comfortable speed. Ten to fifteen infra-red VICON cameras captured the position of retroflective markers, which were placed on the bony landmarks of the child according to the Plug-In-Gait marker model of Vicon (Oxford Metrics, Oxford, UK). Nexus software was used to define gait cycles and estimate joint angles over the three anatomical planes. For children with bilateral CP, both legs were eligible for analysis and for children with unilateral CP, only the affected leg was included. Per included leg, we analyzed the average of two gait trials. After a thorough quality check of all included gait trials, the kinematic parameters reported in literature as well as waveform data were exported using custom-made Matlab software (Mathworks, Inc. version 2014a).

#### Statistical analysis

Only the kinematic joint angle features that were identified during the literature review were considered in the analysis, because good quality kinetic data were not available for all participants. In case the definition of a feature in literature was unclear and we were unable to recalculate it for our own data, it was excluded from the analysis. For each selected feature, a paired samples t-test compared the included group of CP patients pre-treatment versus post-treatment using SPSS Statistics for Windows, version 20.0 (Armonk, NY). The overall probability of making a type I error was maintained at α = 0.05, by adjusting p-values according to the Holm procedure (a stepwise Bonferroni correction) [21–23].

SPM was performed on the time-normalized gait cycles of the lower limb joint angles across the three anatomical planes, taking into consideration the dependency of all points of each gait cycle. A conservative Bonferroni correction of α = 0.01 across the five joints (pelvis, hip, knee, ankle, and foot) maintained a family-wise Type I error rate of 5%.

Firstly, an SPM two-tailed paired t-test compared the mean joint angles of the knee and ankle in the sagittal plane as well as the foot progression angle before and after treatment. A statistical parametric map or SPM{t} was computed, representing the traditional univariate t-statistic being calculated at each point of the gait cycle; this approach is termed ‘mass univariate’. Afterwards, Random Field Theory was used to calculate the critical threshold *t* above which only 1% (α = 0.01) of equally smooth random data samples’ SPM{t} waveforms would be expected to cross[24]. Whenever the experimentally observed SPM{t} exceeded this critical threshold, we computed a supra-threshold cluster probability that indicated a significant statistical difference between the pre-treatment and post-treatment analyses in that part of the gait cycle. A p-value for each supra-threshold cluster was calculated to specify the probability of discovering a cluster with identical temporal breadth when equally smooth random data would be analyzed [24].

Secondly, an SPM paired Hotellings T^2^ was computed to compare the mean joint angles of the pelvis and hip joint before and after BTX-A treatment. The SPM paired Hotellings T^2^ statistic takes into account the time dependency of all points of the gait cycle, as well as the covariance of joint kinematics over the three anatomical planes [25]. Post-hoc t-tests were performed if the Hotellings T^2^ test presented a statistically significant outcome, i.e. when the critical threshold *t* (α = 0.01) was exceeded. These post-hoc t-tests constituted a separate analysis of the pelvis or hip kinematics in the three planes. A full description of this workflow is described in Robinson et al. [13]. All analyses were performed in Python (Python 2.7.2; Enthought Python Distribution, Austin, TX), using open-source SPM1D code (v.0.3; www.spm1D.org).

## Results

### Literature search

**Fig 1:**
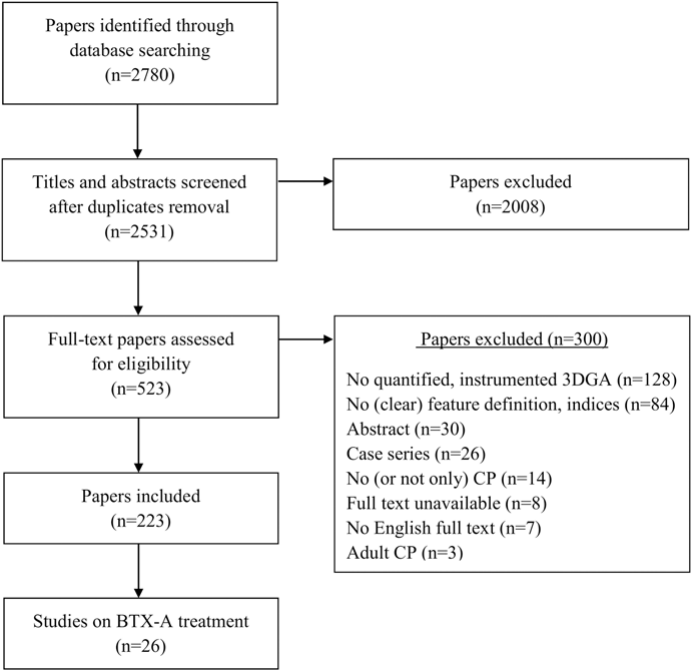
Workflow literature review. This figure describes the workflow which was followed to identify the 26 papers that were included in the literature review. Based on title and abstract, 2008 papers were excluded. After assessing the full-texts, 300 additional papers were excluded using the a priori defined inclusion criteria. In the end, we identified 223 papers that reported on the outcome of treatment in children with CP by means of 3DGA evaluations. Of those 223 papers, 26 reported on the outcome of BTX-A treatment and were included for this study.

**Fig 2:**
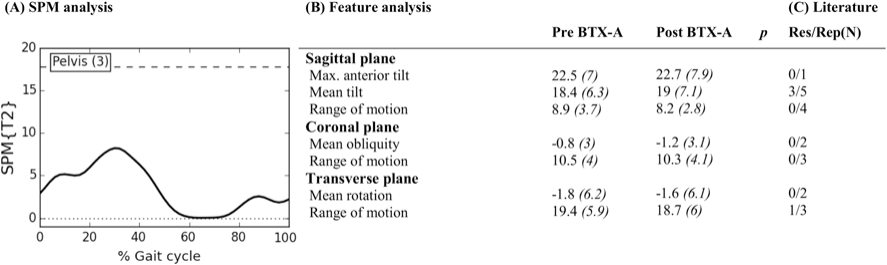
Pelvis across anatomical planes: Mean (°) and *(SD(°))* of kinematic gait features pre- and post-BTX-A treatment (N = 73) compared to SPM analysis (N = 73) and findings from literature review. Panel (A) shows the SPM {T^2^} statistic (α = 0.01) as a function of the gait cycle. The critical threshold (wide dashes) was not exceeded, indicating no significant improvement of BTX-A treatment on the pelvic joint kinematics across the three anatomical planes. Panel (B) shows the mean (°) and (SD) of features extracted from literature. * indicates a significant difference between pre- and post-BTX-A treatment based on Holm’s adjusted p-value (α < 0.05); Max. = maximum. Panel (C) indicates results from literature review. Res/Rep shows the number of papers that reported the feature to be responsive to BTX-A / number of papers that reported the feature.

**Fig 3:**
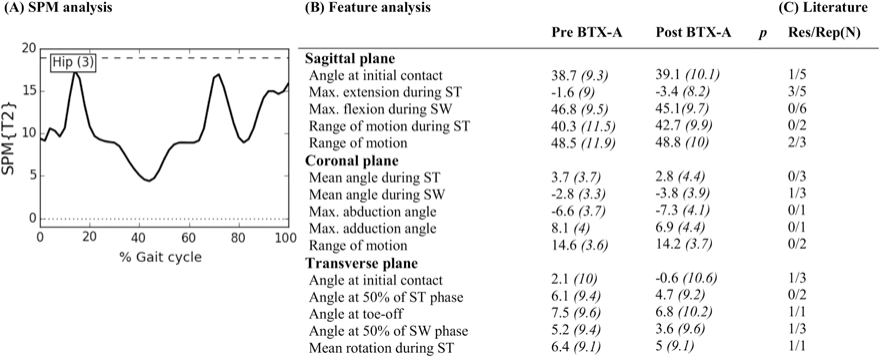
Hip across anatomical planes: Mean (°) and *(SD(°))* of kinematic gait features pre- and post-BTX-A treatment (N = 73) compared to SPM analysis (N = 73) and findings from literature review. Panel (A) shows the SPM {T^2^} statistic (α = 0.01) as a function of the gait cycle. The critical threshold (wide dashes) was not exceeded, indicating no significant improvement of BTX-A treatment on the hip joint kinematics across the three anatomical planes. Panel (B) shows the mean (°) and (SD) of features extracted from literature. * indicates a significant difference between pre- and post-BTX-A treatment based on Holm’s adjusted p-value (α < 0.05); ST = stance; SW = swing; Max. = maximum. Panel (C) indicates results from literature review. Res/Rep shows the number of papers that reported the feature to be responsive to BTX-A / number of papers that reported the feature.

**Fig 4:**
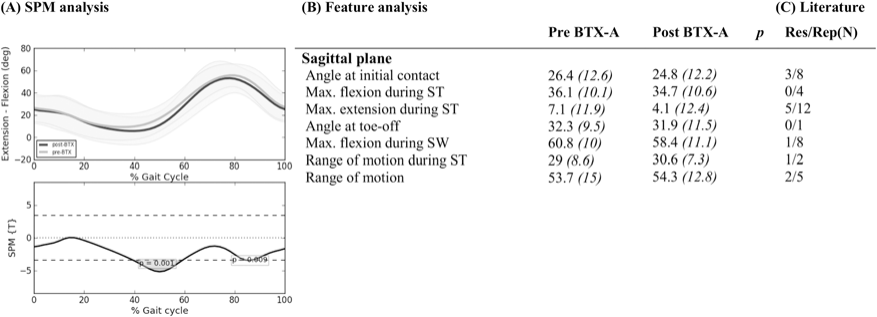
Knee in sagittal plane: Mean (°) and *(SD(°))* of kinematic gait features pre- and post-BTX-A treatment (N = 73) compared to SPM analysis (N = 73) and findings from literature review. Panel (A) shows two graphs. The top graph shows the mean kinematics of the knee in the sagittal plane of 73 included legs pre-BTX-A treatment (light grey) versus post-BTX-A treatment (dark gray). The bottom graph represents the SPM {T} statistic (α = 0.01) as a function of the gait cycle. The critical threshold *t* = 3.425 (wide dashes) was exceeded at 41–59% and at 86% of the gait cycle, indicating a significant improvement of BTX-A treatment on the knee joint kinematics in the sagittal plane. Panel (B) shows the mean (°) and (SD) of features extracted from literature. * indicates a significant difference between pre- and post-BTX-A treatment based on Holm’s adjusted p-value (α < 0.05); ST = stance; SW = swing; Max. = maximum. Panel (C) indicates results from literature review. Res/Rep shows the number of papers that reported the feature to be responsive to BTX-A / number of papers that reported the feature.

**Fig 5:**
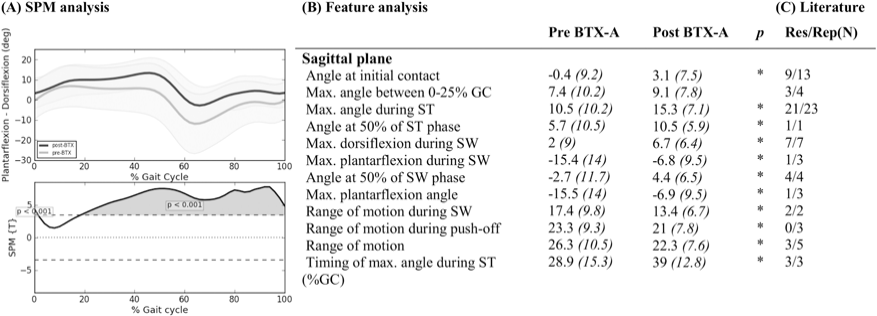
Ankle in sagittal plane: Mean (°) and *(SD(°))* of kinematic gait features pre- and post-BTX-A treatment (N = 73) compared to SPM analysis (N = 73) and findings from literature review. Panel (A) shows two graphs. The top graph shows the mean kinematics of the ankle in the sagittal plane of 73 included legs pre-BTX-A treatment (light grey) versus post-BTX-A treatment (dark gray). The bottom graph represents the SPM {T} statistic (α = 0.01) as a function of the gait cycle. The critical threshold *t* = 3.454 (wide dashes) was exceeded between 0–2% and 22–100% of the gait cycle, indicating a significant improvement of BTX-A treatment on ankle dorsiflexion during the gait cycle. Panel (B) shows the mean (°) and (SD) of features extracted from literature. * indicates a significant difference between pre- and post-BTX-A treatment based on Holm’s adjusted p-value (α < 0.05); GC = gait cycle; ST = stance; SW = swing; Max. = maximum. Panel (C) indicates results from literature review. Res/Rep shows the number of papers that reported the feature to be responsive to BTX-A / number of papers that reported the feature.

**Fig 6:**
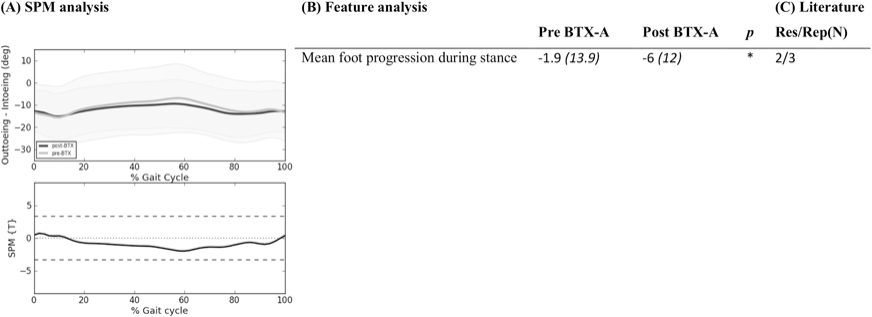
Foot progression angle: Mean (°) and *(SD(°))* of kinematic gait features pre- and post-BTX-A treatment (N = 73) compared to SPM analysis (N = 73) and findings from literature review. Panel (A) shows two graphs. The top graph shows the mean kinematics of the foot progression angle of 73 included legs pre-BTX-A treatment (light grey) versus post-BTX-A treatment (dark gray). The bottom graph represents the SPM {T} statistic (α = 0.01) as a function of the gait cycle. The critical threshold *t* = 3.341 (wide dashes) was not exceeded, indicating no significant effect of BTX-A treatment on the foot progression angle. Panel (B) shows the mean (°) and (SD) of features extracted from literature. * indicates a significant difference between pre- and post-BTX-A treatment based on Holm’s adjusted p-value (α < 0.05). Panel (C) indicates results from literature review. Res/Rep shows the number of papers that reported the feature to be responsive to BTX-A / number of papers that reported the feature.

The literature search yielded a total of 2531 titles and abstracts, which was reduced to a selection of 26 papers that evaluated BTX-A treatment using 3DGA in children with CP (Fig 1)[5], [6], [9], [18], [26–47]. Fifteen papers reported the effect of BTX-A treatment to the gastrocnemius muscle, sometimes in combination with the soleus muscle [5], [6], [26–28], [30], [31], [35], [37–39], [41–43], [46]. Besides BTX-A injections to the gastrocnemius, nine papers also included the hamstrings in a multi-level treatment [9], [18], [29], [33], [34], [36], [40], [44], [47]. Two papers focused solely on BTX-A injections to the hamstrings [32], [45]. The papers reported a median of five features (range 2–49). After features with a similar meaning were grouped together, 53 kinematic features (S1–S5 Tables) and 33 kinetic features (S6–S8 Tables) were identified. Eleven kinematic features, which were ambiguously defined, could not be included in the statistical analysis of the experimental outcome study (part 2). Figs 2–6 list all 42 features that were selected for statistical analysis in the experimental outcome study and show for each of those features the number of papers that have reported it to be responsive to BTX-A treatment in children with CP.

In general, results are mixed. Almost half of all kinematic features were reported only once (n = 12) or twice (n = 12). On the other hand, the maximal dorsiflexion angle during stance was reported in 23 papers and 21 of them found an improved maximal dorsiflexion angle post-BTX-A[5], [6], [9], [18], [26], [27], [29–31], [34–44], [47]. There is also consensus that dorsiflexion in the ankle during the swing phase improves after BTX-A treatment of the gastrocnemius and/or soleus muscle. Seven papers reported an increased maximal dorsiflexion angle during swing[26], [29], [36], [39], [41–43] and four papers reported an increased dorsiflexion angle at 50% of the gait cycle[9], [18], [30], [34]. At the level of the knee, seven out of eight papers, which reported the maximal knee flexion angle during swing, agreed that it was not influenced by BTX-A treatment[5], [6], [9], [18], [31–33], [40]. Knee flexion angle at initial contact and maximal knee extension angle during stance are reported eight[5], [9], [18], [31–33], [40], [47] and twelve[5], [6], [18], [27], [31–33], [37], [40], [42], [44], [45] times respectively, yet results are contradicting. Only three out of eight papers reported a decreased knee flexion angle at initial contact[5], [32], [47] and five out of twelve papers reported an improved knee extension angle during stance[18], [27], [32], [40], [45]. In the hip joint, six papers reported no significant effect of BTX-A injections to the hamstrings and/or gastrocnemius muscles on the maximal hip flexion angle during swing[9], [18], [32], [33], [40], [45].

Of the 33 kinetic features that we identified, 25 were reported only once (n = 17) or twice (n = 8). The features reported most often are the second peak in the ankle moment curve (during the second half of stance phase)[9], [18], [28], [34], [47] and the peak ankle power generation during the gait cycle (maximum positive ankle power)[5], [6], [26], [34], [39], [44], [47]. Three out of five studies agreed that the second peak internal plantarflexion moment increased post-BTX-A[9], [18], [47]. Six out of seven studies that reported the maximal ankle power generation did not change significantly after BTX-A treatment[5], [6], [26], [34], [39], [47].

### Experimental outcome study

A total of 53 patients were included in this study. Patient characteristics are described in Table 1. The majority of patients were diagnosed with bilateral CP (n = 36) and GMFCS level I (n = 25). There was a median of 26.5 days (range 1–91 days) on average between the pre-3DGA and the date of BTX-A treatment. The median time between the BTX-A treatment session and the post-BTX-A 3DGA was 58 days (range 45–114 days). Seventy-three legs were included in statistical analysis.

**Table 1:** Patient Characteristics (N = 53).

**Gender**		
	Female	18
	Male	35
**Diagnosis**		
	Bilateral CP	36
	Unilateral CP	17
**Mean age (at time of pre-3DGA) (years, SD)**		6.1 (2.3)
**Mean weight (at time of pre-3DGA) (kg, SD)**		20.1 (7.0)
**Mean height (at time of pre-3DGA) (cm, SD)**		114.0 (14.6)
**GMFCS**		
	Level I	25
	Level II	17
	Level III	11
**Walking aids during 3DGA**		
	None	43
	Support of one hand	1
	Kayewalker	9

The median dosage of BTX-A injected into multiple sites of the hamstrings was 4U/kg body weight with a range of 2 to 6 U/kg body weight. A median dosage of 4 U/kg body weight was also administered to the gastrocnemius muscle, spread over different sites, with a range of 2 to 7.5 U/kg body weight. Often iliopsoas, adductors, rectus femoris, soleus, and tibialis posterior were also included in the multilevel BTX-A treatment, though less frequently (Table 2). In accordance to the integrated treatment approach, all children received serial stretching casts for the lower and/or upper legs for a period between 1 to 4 weeks. Children with unilateral CP also received casts for both legs with the aim of obtaining a more symmetric gait pattern.

**Table 2:** Muscles treated with BTX-A (N = 73 treated limbs).

Muscles	Number of limbs injected	Median dose U/kg body weight (range)
Iliopsoas *	52	2 (1–3)
Adductors	37	1.5 (1–3)
Rectus femoris	11	1.5 (0.75–2)
Hamstrings	100	4 (2–6)
Gastrocnemius *	73	4 (2–7.5)
Soleus	18	2 (1–3)
Tibialis posterior	5	2 (1.5–2)

* Median dose and range are based on 71 limbs, as dosages were unavailable for two limbs.

Figs 2–6 describe the results for all statistical analyses. For the pelvis and hip joint, neither of the statistical analyses found significant changes. SPM did find significant improvements post-BTX-A-treatment between 41–59% and at 86% of the gait cycle, while the ankle dorsiflexion improved post-BTX-A treatment between 0–2% and 22–100% of the gait cycle. Figs 2–6 also show that out of 42 features, 11 ankle joint features in the sagittal plane and the mean foot progression angle during stance were found to be significantly improved after BTX-A treatment.

## Discussion

In this study, we tested the hypothesis that lower limb joint kinematics of children with a spastic diagnosis of CP would improve toward a more typical gait pattern post-BTX-A. We compared two statistical approaches. On the one hand, we analyzed kinematic gait features that have previously been reported in literature and on the other hand we conducted SPM analyses. In summary, both approaches detected gait changes, mainly at the ankle. Both feature and SPM analyses concluded that no changes in gait occurred at the level of the pelvis and hip. After treatment, based on SPM analysis, we noted a significantly improved knee extension during stance, an earlier peak knee flexion during swing, and an increased ankle dorsiflexion throughout most of the gait cycle. Post-BTX-A, feature analysis also highlighted improved dorsiflexion of the ankle at different time points of the gait cycle, and additionally an increased outtoeing during stance of the foot in the transverse plane, but no effect at the knee. From literature, we learned that results of BTX-A treatment based on feature analyses are generally mixed. Furthermore, feature definitions were not always clear enough to allow us to recalculate them on our own data. For each joint, a more detailed discussion of our results compared to literature is presented below.

### Pelvis and hip

The SPM and feature analyses of the experimental outcome study did not highlight significant effects of BTX-A treatment on the pelvic and hip kinematics in the three anatomical planes. In the same line, few papers in literature report a significant change of pelvic and hip kinematics post-BTX-A treatment. Three out of five studies evaluating ‘mean pelvic tilt in the sagittal plane’, reported a significantly higher anterior tilt after treatment[32], [33], [44]. Corry et al. reported an increased anterior tilt as a source of concern after hamstrings injection if the psoas was left untreated[32]. In the present study, 71% of treated limbs also received psoas injections, hence we did not expect an increased pelvic tilt after BTX-A. In the hip, literature frequently reported on three kinematic features, namely angle at initial contact, maximal hip extension during stance, and maximal hip flexion during swing. Galli et al. evaluated BTX-A injections to the gastrocnemius muscle and reported a slight deterioration towards an increased hip flexion throughout the gait cycle, which emphasizes the need for a multilevel treatment approach[5]. Depending on whether the hip flexors are included in the multilevel BTX-A treatment, the maximal hip extension in stance may significantly improve or not. This was demonstrated by Desloovere et al.[33], Svehlik et al.[44] and also by Papadonikolakis et al. who has reported an increase in maximal hip extension during stance, only in the group of patients who received a multi-level BTX-A treatment[40]. However, it must be noted that neither of these studies attempted to maintain the type I error rate at 0.05 by accounting for the covariance of multiple dependent gait features, so a false positive result cannot be excluded.

### Knee

Compared to the pre-BTX-A condition, SPM analysis of the experimental data noted a significantly improved knee extension and an increased slope towards flexion during terminal stance and pre-swing (between 41% - 59% of the gait cycle). This result may be related to the hamstrings injections. Spasticity of the hamstrings could impede a sufficient amount of knee extension during stance and at the end of swing phase. After BTX-A injection into the hamstrings, a reduction in tone can be achieved; hence improved knee extension during stance can be expected. Additionally, an improvement in knee extension could also be expected after BTX-A injections to the gastrocnemius. On the contrary, no significant differences were found based on the paired samples t-tests on seven gait features of the knee. Is it possible that the two features related to terminal stance and pre-swing, namely ‘maximal knee extension angle during stance’ and ‘knee flexion angle at toe-off’, are insufficient to characterize these changes in the knee motion during gait post-BTX-A. At the level of the knee, changes in gait might be better characterized by considering the terminal stance and pre-swing phase as a whole. SPM analyses of gait phases are easily interpretable and avoid the need of defining additional features while further contributing to the problem of ‘multiple t-testing’. Alternatively, if feature analysis is preferred, we may also conclude that it would be interesting to define an additional feature, for instance knee flexion velocity during pre-swing.

In literature, results are mixed as five out of twelve papers found a significant increase in maximal knee extension during stance[18], [27], [32], [40], [45]. It should be noted that all papers who did not report an improved knee extension during stance either focused the BTX-A treatments solely on the triceps surae[5], [6], [31], [37], [42] or the post-3DGA evaluation was, on average, longer than one year[33], [44].

SPM analysis also indicated a significant change in a short phase of the knee kinematics during mid-swing, possibly indicating a shift in timing of achieving maximal knee flexion during swing. Unfortunately, the definition of ‘amount of delayed knee flexion in swing’, which was reported twice in literature with mixed findings, could not be interpreted and reproduced in our analysis[9], [18]. The only feature during swing that we analyzed was ‘maximal knee flexion angle during swing’, which was found to be unchanged after treatment. In literature, this feature was significantly increased in only one out of eight papers[5]. Galli et al. did not control for an increased type I error risk in their analysis of seventeen kinematic and kinetic features[5]. Nevertheless, their study solely investigated treatment of the gastrocnemius muscle, while the peak knee flexion during swing may be predominantly related to rectus femoris spasticity. In the current study, the average peak knee flexion during swing was around 60°, which is well within the one standard deviation around the mean of our typically developing children. This may explain the fact that the rectus femoris was only injected in 15% of treated limbs. Of the other seven studies that reported no changes in the peak knee flexion during swing, only Papadonikolakis et al. analyzed short-term changes after BTX-A in a patient group of whom some also received rectus femoris injections[40]. However, it is unclear for how many patients the rectus femoris was included.

### Ankle

SPM analysis of the experimental data highlighted a significant increase in dorsiflexion at initial contact after BTX-A treatment. From mid-stance through the remaining part of the gait cycle, the dorsiflexion angle was shown to be significantly increased as well. Features evaluated using paired samples t-tests resembled the results of the SPM analysis. Also in literature, many papers have provided evidence to support these results[5], [6], [9], [18], [26], [27], [29–31], [34–44], [47]. These results were expected as many CP children suffer from gastrocnemius spasticity potentially leading to equinus gait, which is characterized by an abnormal plantarflexion angle throughout the gait cycle. By means of BTX-A injections, the resulting tone reduction facilitates the motion towards dorsiflexion in stance and to clear the foot during swing phase. In the context of an integrated spasticity treatment, the serial stretching casts which were applied during the first weeks after BTX-A might further support this effect. The study of Bottos et al. is the only paper not reporting a short-term beneficial effect of BTX-A on the ankle joint kinematics[28]. However these results are probably due to a limited sample size (BTX-A groep, n = 5 and BTX-A plus casting group, n = 5).

### Foot

At the level of the foot, in contrary to SPM which found no effects, the paired samples t-test on the experimental data revealed a significant improvement of the ‘mean foot progression angle during the stance phase’. Because the motion of one joint is also co-varying (dependent) with the motion at other joints, we applied a Bonferroni correction for the five SPM analyses to obtain a more appropriate alpha level. However, as was reported previously in literature, a Bonferroni correction might be too strict and thus produce an increased chance of false negatives (type II error) [10]. With regard to the feature analysis, we applied a Holm’s correction or stepwise Bonferroni correction for all features to maintain an alpha level of 0.05, with a lower chance of false negatives (higher power) [21], [22], which might explain the different outcomes between both analysis approaches. Compared to literature, one study by Desloovere et al. did not find a significant effect on the foot progression angle[33]. However, the post-3DGA evaluation in this study was on average one year and ten months post-BTX-A injections, making it unlikely to detect a therapeutic effect from the treatment. Two other studies also reported an increased outtoeing after BTX-A[9], [18]. They both performed Bonferroni corrections, making a false positive result unlikely. In these two studies, as well as in our experimental study, all children received injections to the gastrocnemius along with a period of stretching casts to the lower legs. Hence more gait improvements can be expected in the distal joints such as the ankle and foot.

### Literature

Apart from different statistical approaches and whether or not the Type I error is controlled, another factor that could account for differences in results among the included papers could be the small sample sizes. With twelve papers evaluating a patient group of less than 20 patients, it is possible that a bias was present in the selected experimental population[5], [6], [27], [28], [31], [32], [34], [37], [44–47]. The timing of the post-BTX-A 3DGA ranged from two weeks to more than one year across the included papers. This may result in heterogeneous conclusions, given the limited time frame in which BTX-A injections are effective and the importance to acknowledge the effect of other parameters, such as age, on gait when timing of follow-up increases. Naturally, the different clinical characteristics of experimental patient groups as well as the diversity regarding the different muscles which were treated and the dosage of BTX-A, may also contribute to different outcomes. However a detailed analysis of all these factors would require a thorough evaluation of the methodological quality of the papers, which was beyond the scope of the current study.

### Limitations

Limitations of the current study need to be addressed. While creating an overview of the features from literature, rather subjective judgments were made on whether or not particular features could be merged. To avoid bias as much as possible all features were first independently judged by two reviewers, after which a third reviewer was consulted in cases of disagreement. This task was also complicated because a number of features were not clearly defined and thus not easily recalculated with our own data. Consequently, we were unable to include all reported features in the outcome study. Another limitation was the study population, which was recruited from the retrospective database of the University Hospital Pellenberg, Belgium, where every child is treated according to the same rehabilitation guidelines. As a consequence, generalization of results is limited and could be improved through multi-center studies. It was reported by Molenaers et al. that the first two BTX-A treatments are most effective in increasing function[19]. In our intervention study, thirteen patients who had received a third BTX-A treatment and one who had received a fourth treatment were included, which could have led to a reduced effect of BTX-A compared to earlier studies. A sample selection, only containing children after their first or second BTX-A treatment was not always possible because children were often too young for a 3DGA when they were receiving their first treatments.

## Conclusion

In conclusion, both the outcome of the current study as well as literature reports conclude that BTX-A injections are a valuable treatment option to improve gait in children with CP. The effects were mainly observed at the ankle joint and to a lesser extent at the knee. Different results can be explained by various factors which have been illustrated in this study. The key issue which we discussed in this study was the statistical analysis used to analyze 3DGA data. In literature, the effect on specific gait features is traditionally reported. Considering that over half of all extracted features were only reported once or twice for a list of 26 studies, it can be concluded that there is no consensus on which features should be evaluated to assess the effect of BTX-A on the gait pattern of children with CP. Additionally, the risk of obtaining false positive results (Type I error) quickly increases when multiple dependent gait features are analyzed and attempts to control this risk using a Bonferroni correction were in turn decreasing power (increasing the chance of Type II error).

Our study compared this frequently reported feature analysis to SPM. Our findings suggest that both statistical methods might be appropriate to analyze kinematic and kinetic data to examine the effect of BTX-A on gait. However, we suggest that a clear, definite hypothesis should be stated a priori and an adequate, statistical approach should be selected to accompany this hypothesis. When an analysis of features is preferred following a specific hypothesis, we note that alternatives to the Bonferroni correction are available to deal with the risk of making a Type I error[10], [21], [22]. The currently presented literature review could be a guide for feature selection. When reporting features, care should be taken for an unambiguous feature definition that is clinically meaningful. For example ‘maximal dorsiflexion during the gait cycle’ could, depending on the clinical presentation of the child, occur during loading response, at the beginning of the third rocker, or during swing phase and might thus not be very meaningful to a clinician.

If it is difficult to specifically hypothesize on the direction and magnitude of changes post-treatment, SPM analysis might be preferred. SPM analysis allows you to analyze kinematic and kinetic waveforms as a whole, or particular gait phases, e.g. the swing phase for the knee joint. It has the advantage of making a priori data reduction redundant and taking into account the covariance of all points of the gait cycle. Nevertheless, at this time, it is not possible to robustly address the co-variation of different joints (e.g. knee and ankle).

For this study, we chose to illustrate the value of SPM by analyzing the effect of BTX-A treatment on gait because treatment protocols are well standardized and its effect has often been reported in literature. Furthermore, the effect of BTX-A is evaluated rather quickly after treatment, ensuring little impact of other treatments or other factors such as age that may influence gait. However, it would also be interesting to analyze the effects of other treatments in children with CP, such as selective dorsal rhizotomy or orthopedic surgery using SPM. SPM might even be more appropriate for pathologies where kinematic and kinetic gait deviations post-treatment have not yet been routinely recognized by clinicians. Non-directed hypotheses evaluated with SPM analysis may help to reduce the wealth of 3DGA data and aid the construction of more specific, directed hypotheses in the future.

## Supporting Information

S1 PRISMA Checklist (PDF)

S1 TREND Checklist (PDF)

S1 Table Kinematic pelvic features extracted from 26 papers, identified through systematic literature search. (PDF)

S2 Table Kinematic hip features extracted from 26 papers, identified through systematic literature search. (PDF)

S3 Table Kinematic knee features extracted from 26 papers, identified through systematic literature search. (PDF)

S4 Table Kinematic ankle features extracted from 26 papers, identified through systematic literature search. (PDF)

S5 Table Kinematic features of the foot in the transverse plane extracted from 26 papers, identified through systematic literature search. (PDF)

S6 Table Kinetic hip features extracted from 26 papers, identified through systematic literature search. (PDF)

S7 Table Kinetic knee features extracted from 26 papers, identified through systematic literature search. (PDF)

S8 Table Kinetic ankle features extracted from 26 papers, identified through systematic literature search. (PDF)
