# Supplementary material for: Literature Review and Comparison of Two Statistical Methods to Evaluate the Effect of Botulinum Toxin Treatment on Gait in Children with Cerebral Palsy
Source: PLoS One. 2016 Mar 31;11(3):e0152697. doi: 10.1371/journal.pone.0152697 (PMC4816309; doi:10.1371/journal.pone.0152697)
Supplement: S8 Table — (PDF) [file pone.0152697.s010.pdf]

S8 Table. Kinetic ankle features extracted from 26 papers, identified through systematic literature search

	[1]	[2]	[3]	[4]	[5]	[6]	[7]	[8]	[9]	[10]	[11]	[12]	[13]	[14]	[15]	[16]	[17]	[18]	[19]	[20]	[21]	[22]	[23]	[24]	[25]	[26]
1st max. PF moment (1st peak before MSt)			**x**							**x**							**x**									**v**
PF moment at the end of 1st DS phase																**v**										
PF moment at LR								**v**									**v**									
2nd max. PF moment (2nd peak before TO)			**x**					**v**		**x**							**v**									**v**
PF moment at begin of 2nd DS phase																**x**										
Max. PF moment in Mst - TSt								**v**																		
Max. moment																									**x**	
Timing of max. moment (% of GC)																									**v**	
M index											**v**															
M area											**v**															
AMQ					**v**											**x**										**v**
APQ					**v**											**x**										**v**
Max. power generation during GC	**x**									**x**		**x**				**x**						**v**			**x**	**x**
Max. generation in mid-stance																									**v**	**v**
Power generation at pre-swing								**x**									**x**									
Max. power absorption in early stance										**x**		**v**				**x**										
Power absorption at LR								**v**									**v**									
Generated work										**x**	**x**															
Absorbed work										**v**	**x**															

V = Significantly changed post-BTX-A treatment; X = Not significantly changed post-BTX-A treatment; Max. = Maximal; PF = plantarflexion; MSt = mid-stance; DS = double support; LR = loading response; TO = toe-off; TSt = terminal stance; GC = gait cycle; M index = equation 1 in [11]; M area = area beneath moment curve; AMQ = 1st moment peak / 2nd moment peak; APQ = 1st power generation peak / 2nd power generation peak.

References

[1] J. D. Ackman, B. S. Russman, S. S. Thomas, C. E. Buckon, M. D. Sussman, P. Masso, et al. “Comparing botulinum toxin A with casting for treatment of dynamic equinus in children with cerebral palsy.,” *Dev. Med. Child Neurol.*, vol. 47, no. 9, pp. 620–7, Sep. 2005.

[2] M. S. Bang, S. G. Chung, S. B. Kim, and S.-J. Kim, “Change of dynamic gastrocnemius and soleus muscle length after block of spastic calf muscle in cerebral palsy.,” *Am. J. Phys. Med. Rehabil.*, vol. 81, no. 10, pp. 760–4, Oct. 2002.

[3] M. Bottos, M. G. Benedetti, P. Salucci, V. Gasparroni, and S. Giannini, “Botulinum toxin with and without casting in ambulant children with spastic diplegia: A clinical and functional assessment,” *Dev. Med. Child Neurol.*, vol. 45, no. 11, pp. 758–762, 2003.

[4] R. N. Boyd, J. E. A. Graham, G. R. Nattrass, and H. K. Graham, “Medium-term response characterisation and risk factor analysis of botulinum toxin type A in the management of spasticity in children with cerebral palsy,” *Eur. J. Neurol.*, vol. 6, no. 4, pp. S37–S45, Nov. 1999.

[5] R. N. Boyd, V. Pliatsios, R. Starr, R. Wolfe, and H. K. Graham, “Biomechanical transformation of the gastroc-soleus muscle with botulinum toxin A in children with cerebral palsy.,” *Dev. Med. Child Neurol.*, vol. 42, no. 1, pp. 32–41, 2000.

[6] I. S. Corry, A. P. Cosgrove, C. M. Duffy, S. McNeill, T. C. Taylor, and H. K. Graham, “Botulinum toxin a compared with stretching casts in the treatment of spastic equinus: A randomised prospective trial,” *J. Pediatr. Orthop.*, vol. 18, no. 3, pp. 304–311, 1998.

[7] I. S. Corry, A. P. Cosgrove, C. M. Duffy, T. C. Taylor, and H. K. Graham, “Botulinum toxin A in hamstring spasticity,” *Gait Posture*, vol. 10, no. 3, pp. 206–210, 1999.

[8] K. Desloovere, G. Molenaers, I. Jonkers, J. De Cat, L. De Borre, J. Nijs, et al. “A randomized study of combined botulinum toxin type A and casting in the ambulant child with cerebral palsy using objective outcome measures.,” *Eur. J. Neurol.*, vol. 8 Suppl 5, pp. 75–87, 2001.

[9] K. Desloovere, G. Molenaers, J. De Cat, P. Pauwels, A. Van Campenhout, E. Ortibus, et al. “Motor function following multilevel botulinum toxin type A treatment in children with cerebral palsy,” *Dev. Med. Child Neurol.*, vol. 49, no. 1, pp. 56–61, 2007.

[10] C. Detrembleur, T. M. Lejeune, A. Renders, and P. Y. K. Van Den Bergh, “Botulinum toxin and short-term electrical stimulation in the treatment of equinus in cerebral palsy.,” *Mov. Disord.*, vol. 17, no. 1, pp. 162–9, Jan. 2002.

[11] M. Galli, M. Crivellini, G. C. Santambrogio, E. Fazzi, and F. Motta, “Short-term effects of ‘botulinum toxin A’ as treatment for children with cerebral palsy: Kinematic and kinetic aspects at the ankle joint,” *Funct. Neurol.*, vol. 16, no. 4, pp. 317–323, 2001.

[12] M. Galli, V. Cimolin, E. M. Valente, M. Crivellini, T. Ialongo, and G. Albertini, “Computerized gait analysis of botulinum toxin treatment in children with cerebral palsy.,” *Disabil. Rehabil.*, vol. 29, no. 8, pp. 659–64, Apr. 2007.

[13] R. M. Kay, S. A. Rethlefsen, A. Fern-Buneo, T. A. L. Wren, and D. L. Skaggs, “Botulinum toxin as an adjunct to serial casting treatment in children with cerebral

palsy," *J. Bone Jt. Surg. - Ser. A*, vol. 86, no. 11, pp. 2377–2384, 2004.

[14] M. C. M. Klotz, S. I. Wolf, D. Heitzmann, S. Gantz, F. Braatz, and T. Dreher, "The influence of botulinum toxin a injections into the calf muscles on genu recurvatum in children with cerebral palsy pediatrics," *Clin. Orthop. Relat. Res.*, vol. 471, no. 7, pp. 2327–2332, 2013.

[15] J. H. Lee, I. Y. Sung, J. Y. Yoo, E. H. Park, and S. R. Park, "Effects of different dilutions of botulinum toxin type A treatment for children with cerebral palsy with spastic ankle plantarflexor: a randomized controlled trial.," *J. Rehabil. Med.*, vol. 41, no. 9, pp. 740–5, Sep. 2009.

[16] D. Metaxiotis, A. Siebel, and L. Doederlein, "Repeated botulinum toxin A injections in the treatment of spastic equinus foot.," *Clin. Orthop. Relat. Res.*, no. 394, pp. 177–85, Jan. 2002.

[17] G. Molenaers, K. Desloovere, J. De Cat, I. Jonkers, L. De Borre, P. Pauwels, et al. "Single event multilevel botulinum toxin type A treatment and surgery: similarities and differences.," *Eur. J. Neurol.*, vol. 8 Suppl 5, pp. 88–97, Nov. 2001.

[18] A. S. Papadonikolakis, M. D. Vekris, A. V Korompilias, J. P. Kostas, S. E. Ristanis, and P. N. Soucacos, "Botulinum A toxin for treatment of lower limb spasticity in cerebral palsy: Gait analysis in 49 patients," *Acta Orthop. Scand.*, vol. 74, no. 6, pp. 749–755, 2003.

[19] F. Polak, R. Morton, C. Ward, W. A. Wallace, L. Doderlein, and A. Siebel, "Double-blind comparison study of two doses of botulinum toxin A injected into calf muscles in children with hemiplegic cerebral palsy.," *Dev. Med. Child Neurol.*, vol. 44, no. 8, pp. 551–5, Aug. 2002.

[20] D. H. Sutherland, K. R. Kaufman, M. P. Wyatt, and H. G. Chambers, "Injection of botulinum A toxin into the gastrocnemius muscle of patients with cerebral palsy: A 3-dimensional motion analysis study," *Gait Posture*, vol. 4, no. 4, pp. 269–279, 1996.

[21] D. H. Sutherland, K. R. Kaufman, M. P. Wyatt, H. G. Chambers, and S. J. Mubarak, "Double-blind study of botulinum A toxin injections into the gastrocnemius muscle in patients with cerebral palsy," *Gait Posture*, vol. 10, no. 1, pp. 1–9, 1999.

[22] M. Svehlik, T. Kraus, G. Steinwender, E. B. Zwick, M. Ladecky, Z. Szabo,et al. "Repeated Multilevel Botulinum Toxin A Treatment Maintains Long-Term Walking Ability in Children with Cerebral Palsy," *Ces. A Slov. Neurol. A Neurochir.*, vol. 75, no. 6, pp. 737–741, 2012.

[23] N. S. Thompson, R. J. Baker, A. P. Cosgrove, I. S. Corry, and H. K. Graham, "Musculoskeletal modelling in determining the effect of botulinum toxin on the hamstrings of patients with crouch gait," *Dev. Med. Child Neurol.*, vol. 40, no. 9, pp. 622–625, 1998.

[24] J. Wissel, J. Muller, A. Baldauf, S. C. Ung, J. P. Ndayisaba, B. Stockl, et al."Gait analysis to assess the effects of botulinum toxin type A treatment in cerebral palsy: an open-label study in 10 children with equinus gait pattern," *Eur. J. Neurol.*, vol. 6, no. 4, pp. S63–S67, Nov. 1999.

[25] E. B. . Zwick, M. . b Švehlík, G. . Steinwender, and W. E. . Linhart, "Short-term effects of botulinum toxin A and serial casting on triceps surae muscle length and equinus gait in children with cerebral palsy," *Ces. a Slov. Neurol. a Neurochir.*, vol. 72, no. 6, pp. 553–558, 2009.

[26] A. W. Zurcher, G. Molenaers, K. Desloovere, and G. Fabry, "Kinematic and kinetic evaluation of the ankle after intramuscular injection of botulinum toxin A in children with cerebral palsy," *Acta Orthop. Belg.*, vol. 67, no. 5, pp. 475–480, 2001.
